# Correlates of tobacco quit attempts and cessation in the adult population of India: secondary analysis of the Global Adult Tobacco Survey, 2009–10

**DOI:** 10.1186/1471-2458-13-263

**Published:** 2013-03-22

**Authors:** Swati Srivastava, Sumit Malhotra, Anthony D Harries, Pranay Lal, Monika Arora

**Affiliations:** 1 Public Health Foundation of India, New Delhi, India; 2 All India Institute of Medical Sciences, New Delhi, India; 3 International Union Against Tuberculosis and Lung Disease (The Union), Paris, France; 4 Department of Infectious and Tropical Diseases, London School of Hygiene and Tropical Medicine, London, UK; 5 International Union Against Tuberculosis and Lung Disease (The Union), South-East Asia Regional Office, New Delhi, India

**Keywords:** Tobacco, Cessation, Health system, India

## Abstract

**Background:**

Nearly 275 million adults (15 years and above) use tobacco in India, which contributes substantially to potentially preventable morbidity and mortality. There is good evidence from developed country settings that use of tobacco cessation services influences intention to quit, with a higher proportion of attempts being successful in fully quitting. There is little evidence about cessation and quitting behaviour in the Indian context. This study assesses the socio-demographic characteristics and cessation services used by adults i) who attempted to quit smoked and smokeless tobacco and ii) who were successful in quitting.

**Methods:**

The study was a cross-sectional secondary data analysis of the Global Adult Tobacco Survey, India, 2009–10. There were 25,175 ever tobacco users aged 21 years and above included in the study. Bivariate and multivariate logistic regression analysis was done to determine associations between socio-demographic variables and cessation services utilized with attempts to quit tobacco and successful quitting.

**Results:**

Of the ever tobacco users, 10,513 (42%) made an attempt to quit tobacco, and of these 4,395 (42%) were successful. Significant associations were demonstrated between male gender, increasing educational attainment and higher asset quintiles for both those who attempted to quit and those who were successful. Younger age groups had higher odds of quit attempts than all except the oldest age group, but also had the lowest odds of successful quitting. Heath care provider advice was positively associated with attempts to quit, but both advice and use of cessation aids were not associated with successful quitting.

**Conclusions:**

This study provides the first national evidence on the relationships between quitting attempts and successful quitting with socio-demographic characteristics, health care provider advice and use of cessation services. The findings of the study have important implications for scaling up tobacco cessation services in India, and indicate a need to re-examine in greater detail the effects of socio-demographic factors, type of tobacco product used and levels of dependency on quitting. Health system factors such as coverage and accessibility of cessation services, type of service, and its duration and follow up also have to be examined in detail to ascertain effects on quitting behavior.

## Background

More than one third of India’s adult population (275 million persons) was estimated to be tobacco users in 2010 [1]. These persons comprised 68.9 million smokers (defined as persons smoking cigarettes, bidis, hukkahs, cigars and pipes), 163.7 million smokeless users (defined as persons chewing smokeless tobacco) and 42.3 million users of both products [1]. Tobacco inflicts high costs on society, with total direct and indirect costs estimated to be US$1.7bn in India in 2004 [2]. The nationally representative Million Deaths Study in India showed that smoking in adults aged 30 to 69 years resulted in approximately 1 in 20 deaths of women and 1 in 5 deaths of men in the country; with smokers having an all-cause mortality 1.7 times in males and twice in females, compared to nonsmokers of the same gender [3]. Smokeless tobacco, including tobacco with betel leaf is associated with a high risk of oral cancer in India [4]. The gender gradient is also evident in consumption patterns, with 24% of males and 3% of females in the country being users of smoked products; and 33% of males and 18% of females being users of smokeless products [1]. Cultural norms do not permit women to smoke, but there are no restrictions on chewing tobacco, accounting for the high chewing prevalence [5]. Socioeconomic status is strongly related to tobacco use, and socio-economic differences contribute substantially to inequalities in tobacco-attributable mortality and morbidity [6-8]. Getting smokers to quit the habit leads to a reduction in morbidity and mortality, and is a worthwhile intervention [9-11].

It has been reported that availability of tobacco cessation services, the methods of cessation support, health care provider (HCP) involvement, and pharmacotherapy play a crucial role in successful attempts to quit smoking [12,13]. The Government of India initiated the National Tobacco Control Programme in 2007–08 as the first national program for tobacco control. The program mandates implementation of different tobacco control initiatives at the national, state and district levels, encompassing law enforcement, awareness campaigns, training, and monitoring and evaluation, including surveillance [1]. Another component of the program is to provide tobacco cessation services in the primary health care system [1]. The district is the basic administrative unit for many public health services in the country and is responsible for the development of tobacco cessation services (with the state’s technical support) in general hospitals, TB hospitals, regional cancer centres, teaching colleges and other institutions [1]. The Government of India in collaboration with the World Health Organization established twenty-one tobacco cessation clinics from the years 2001 to 2009, under the Tobacco Free Initiative to provide tobacco cessation services [14]. However, the coverage and effectiveness of these cessation services have yet to be ascertained.

The 2009–10 Global Adult Tobacco Survey (GATS), the first ever nationally-representative survey of key tobacco indicators in India, reported that in the last twelve months, 38% of tobacco users made attempts to quit, 47% visited a health care-provider for cessation, and 46% were advised to quit by a HCP [1]. However, there is very little detailed information in India on the socio-demographic characteristics of people who attempt to quit tobacco, what services they accessed and whether or not they successfully quit. This needs to be further explored to better understand and implement effective tobacco cessation services within the country. The aim of the current study was to assess the socio-demographic characteristics and cessation services used by adults i) who attempted to quit smoked and smokeless tobacco and ii) who were successful in quitting.

## Methods

### Study design

The study was a cross-sectional secondary data analysis of the Global Adult Tobacco Survey, India, 2009–10 [1].

### Setting

India is a large country with a total population of 1.2 billion [15]. There are 29 states and six union territories. Following market based economic reforms in 1991, India became one of the fastest growing economies of the world and today is the world’s tenth largest economy by nominal Gross Domestic Product (in 2011, estimated at USD$1,676 billion) [16]. The human sex ratio is estimated at 940 females per 1000 males [17]. Despite impressive economic growth in the last decade, India has the largest concentration of people living below the poverty line [16]. India has a universal health care system provided by the public sector [17]. There is a large private sector in both rural and urban areas, with the public sector providing treatment for only an estimated 26% of illness episodes [18].

The original data of GATS were collected from June 2009 to January 2010 in a household survey by the International Institute for Population Sciences, Mumbai, with technical support from Centers for Disease Control, Atlanta, the World Health Organization and RTI International [1]. The GATS was conducted with the objectives of measuring the impact of tobacco control efforts of the Cigarettes and Other Tobacco Products Act, 2003, and to systematically monitor adult tobacco indicators. A nationally representative sample was drawn from the 29 states and two out of the six Union Territories covering almost 99.9% of the population of the country. The survey included structured interviews with 69,296 respondents aged 15 years and above.

### Participants

The original GATS survey used probability proportional to size (PPS) sampling, separately at urban and rural levels. This current study includes a total number of 61,664 adult men and women aged 21 years and above, from both rural and urban areas of India. This age group was selected because only 4.5% of those younger (15–20 years) in the survey ever used any type of tobacco products, while 19.7% of those 21 years or older used tobacco. The 15–20 year olds might not have yet initiated tobacco use but may do so at a later stage, and including this age group was thought to bias the results. The prevalence of tobacco use in both men (48%) and women (20%) is high, and therefore both are included in the analysis.

### Variables

The exposure variables used for assessing associations were:- age group, place of residence (urban or rural), gender (male or female), educational attainment (no formal education, up to primary education, up to secondary education, or higher education), asset quintile (lowest, lower, middle, higher, and highest), work status (worked in the last twelve months or not), advice by a HCP to quit tobacco, and use of tobacco cessation aids. The variable called “asset quintile” was created using a summative score of inverse weighted proportions of possession of the following assets- electricity, flush toilet, car, moped/scooter/motorcycle, television, refrigerator, washing machine, fixed telephone, cell-phone, and radio. The summative score was then divided into quintiles to obtain asset quintiles, which were used as a proxy for wealth. This methodology has been validated elsewhere [19].

The outcome variables were attempts to quit tobacco use (defined as tobacco users who have reported that they tried to stop smoking and/or chewing tobacco at any time in the past twelve months for current users, or those who had successfully quit more than twelve months before the survey) and quit tobacco (defined as tobacco users who have stopped smoking and/or chewing at any time in the past) [1]. For current users, an attempt to quit was defined as making an attempt to quit tobacco in the last twelve months in the source survey. For former users, attempts to quit tobacco were asked for the entire duration of their tobacco use. We created another category of tobacco users, called ever tobacco users, comprising all respondents (current and former) who have ever used any form of tobacco in their life.

Using the original dataset of GATS (where the data were already collected in 2009 and 2010), a working electronic database was extracted using STATA version 11.2 software (Statacorp, College Station, Texas, USA) [20]. The dataset is available for public use from the United States Centers for Disease Control and Prevention website [21].

### Analysis and statistics

The analysis of the extracted data was conducted using STATA version 11.2 software [20]. First, a descriptive analysis for all variables included in the study was done. Second, an initial bivariate analysis was done by searching for associations of exposure variables with the outcome variables. After obtaining initial level associations, stepwise multiple logistic regression analysis with forward selection was performed for finding associations and adjusting for individual level variables which had been found to be significant. Significance levels for both the initial bivariate analysis and multiple logistic regression analysis were set at 0.05.

### Ethics approval

Ethics approval for the study was obtained by the Ethics Advisory Group of the International Union Against Tuberculosis and Lung Disease (The Union), Paris (EAG number: 120/12) and the Institutional Ethics Committee of the Public Health Foundation of India, New Delhi (TRC-IEC-142/12).

## Results and discussion

**Figure 1:**
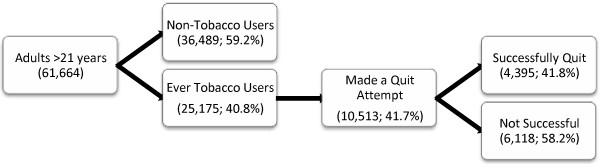
Tobacco use trajectory of study participants based on Global Adult Tobacco Survey India data, 2009–10.

Figure 1 shows the tobacco use trajectory of adults greater than 21 years from the GATS India survey data. Of the 25,175 ever tobacco users aged 21 years and above, 10,513 (42%) made an attempt to quit tobacco and, of these, 4,395 (42%) were successful. It is important to note that individuals might have made more than one successful or unsuccessful attempt to quit tobacco, for either smoked or chewing tobacco.

### Attempts to quit smoked and/or chewing tobacco

Table 1 presents the socio-demographic characteristics of ever tobacco users who attempted to quit tobacco compared with those who did not attempt to quit. On bivariate analysis, tobacco users in the 21–30 years age group were found to have made marginally higher attempts to quit as compared to the oldest age group (>50 years old), however this association was not statistically significant. All other age groups had lesser odds for attempts to quit as compared to the oldest age group. Persons belonging to the 41–50 years age group had a 0.83 times lesser odds of intending to quit compared to the 50+ age group, adjusting for all other covariates (95% Confidence Interval (CI) 0.73-0.93, p <0.01). Male tobacco users had 1.48 times higher bivariate odds ratio for attempts to quit than females (95% CI 1.36-1.62, p <0.001). Even after adjusting for other covariates, male gender was associated with higher odds for attempting to quit tobacco.

**Table 1:** Socio-demographic characteristics and use of cessation services amongst tobacco users who attempted and did not attempt to quit

**Covariates**	**Attempted quitting N = 10,513**	**No attempts to quit N = 14,662**	**Bivariate odds ratios**	**Adjusted odds ratios**
[1]	[2]	[3]	[4]	[5]
**Age group**				
21 to 30 years	2,348 (26)	3,398 (25)	1.02 [0.90-1.14]	0.94 [0.83-1.06]
31 to 40 years	3,048 (27)	4,623 (27)	0.93 [0.84-1.03]	0.87 [0.78-0.97]*
41 to 50 years	2,247 (19)	3,223 (21)	0.87 [0.78-0.98]*	0.83 [0.73-0.93]**
Greater than 50 years	2,870 (28)	3,418 (27)	1.00	1.00
**Gender**				
Female	2,847 (25)	4,802 (33)	1.00	1.00
Male	7,666 (75)	9,860 (67)	1.48 [1.36-1.62]***	1.27 [1.15-1.40]***
**Educational attainment**				
No formal education	3,212 (37)	5,278 (45)	1.00	1.00
Up to primary	2,943 (28)	4,254 (28)	1.24 [1.12-1.37]***	1.14 [1.02-1.27]*
Up to secondary	3,489 (29)	4,220 (23)	1.61 [1.45-1.78]***	1.45 [1.28-1.64]***
Higher education	845 (6)	858 (4)	2.04 [1.67-2.48]***	1.77 [1.41-2.22]***
**Work status**				
Not working	7,313 (67)	10,572 (70)	1.00	1.00
Working	3,178 (33)	4,048 (30)	1.14 [1.04-1.24]**	1.05 [0.96-1.15]
**Asset quintile**				
Lowest	2,873 (36)	4,514 (41)	0.60 [0.52-0.70]***	0.82 [0.68-0.98]*
Lower	2,344 (24)	3,318 (24)	0.70 [0.59-0.82]***	0.88 [0.73-1.06]
Middle	2,196 (19)	2,959 (17)	0.75 [0.64-0.89]***	0.90 [0.75-1.08]
Higher	1,839 (13)	2,271 (12)	0.79 [0.66-0.95]*	0.87 [0.72-1.05]
Highest	1,261 (8)	1,600 (6)	1.00	1.00
**Place of residence**				
Urban	3,406 (23)	4,399 (21)	1.00	1.00
Rural	7,107 (77)	10,263 (79)	0.88 [0.81-0.95]**	1.07 [0.97-1.17]
**Health Care Provider (HCP) advice**				
Not advised to quit tobacco by a HCP	8,340 (80)	12,862 (88)	1.00	1.00
Advised to quit tobacco by a HCP	2,173 (20)	1,800 (12)	1.81 [1.62-2.02]***	1.79 [1.60-2.00]***
**Cessation aid used**				
No cessation aid used	9,267 (89.1)	14,662 (100)	-	-
Counseling/Cessation clinic	804 (6.82)	-	-	-
Nicotine replacement therapy	108 (0.74)	-	-	-
Prescription medication	98 (0.72)	-	-	-
Ayurvedic, Homeopathic, Unani Medicines^i^	310 (1.16)	-	-	-
Quitline	56 (0.56)	-	-	-

Legend:

Columns [2] and [3] show descriptive statistics of the individual sample from the Indian Global Adult Tobacco Survey (GATS) 2009–10 data, showing the sample (N) and survey-weighted frequency (%) of attempts to Quit Tobacco among ever tobacco users.

Columns [4] and [5] show bivariate and fully adjusted odds ratios with 95% confidence intervals (ORs and 95% CI), respectively, from logistic regression analysis of attempts to quit tobacco compared to no attempts based on 25,175 ever tobacco user adults from the Indian Global Adult Tobacco Survey (GATS) 2009–10 data.

^i^Ayurvedic, Homeopathic, Unani are traditional medicine practices widely used in India.

***p < 0.001, ** p < 0.01, * p < 0.05.

Educational attainment was progressively associated with increased attempts to quit. Compared to those with no formal education, the adjusted odds ratio (AOR) of making a quit attempt in those with education up to primary level was 1.14 (95% CI 1.02-1.27, p <0.05), in those with education up to secondary level was 1.45 (95% CI 1.45-1.64, p <0.001), and in those with higher education was 1.77 (95%CI 1.41-2.22, p <0.001). In the bivariate analysis, there was a progressive gradient in the odds of attempting to quit by asset quintile, but none of the lower quintiles reached the attempted quitting levels seen in the highest quintile. In the fully adjusted model the lowest asset quintile had an AOR for attempting to quit 0.82 times that of the highest quintile (95% CI 0.68-0.98, p <0.05). Those who were advised to quit tobacco by a HCP had a bivariate odds ratio for attempts to quit 1.81 times more than those who were not advised (95% CI 1.62-2.02, p <0.001). In the fully adjusted model the AOR was 1.79 and remained significant (95% CI 1.60-2.00, p <0.001). Of those attempting to quit, approximately 10% used a cessation aid, the main aid used being any form of counseling.

### Successful quitting

Table 2 shows the socio-demographic characteristics and cessation services utilization amongst tobacco users who were either successful or unsuccessful in quitting tobacco. Compared to the 50+ years age group, the adjusted odds of successfully quitting tobacco were lower in the younger age groups. In contrast to the model for attempts to quit, males had an odds of quitting that was 0.78 times that in females (95% CI 0.67-0.92, p <0.01). A similar gradient as for the model for attempts to quit was observed across the asset quintiles. The AOR for quitting was lower in the lowest, lower and middle quintiles.

**Table 2:** Socio-demographic characteristics and use of cessation services amongst tobacco users attempting to quit who were successful and not successful

**Covariates**	**Successfully quit N = 4,395**	**Not successful N = 6,118**	**Bivariate odds ratios**	**Adjusted odds ratios**
[1]	[2]	[3]	[4]	[5]
**Age group**				
21 to 30 years	857 (22)	1,491 (28)	0.50 [0.42-0.60]***	0.47 [0.38-0.56]***
31 to 40 years	1,092 (23)	1,956 (29)	0.50 [0.42-0.58]***	0.48 [0.41-0.57]***
41 to 50 years	912 (19)	1,335 (20)	0.62 [0.52-0.74]***	0.62 [0.52-0.74]**
Greater than 50 years	1,534 (36)	1,336 (23)	1.00	1.00
**Gender**				
Female	1,216 (27)	1,631 (23)	1.00	1.00
Male	3,179 (73)	4,487 (77)	0.78 [0.68-0.90]***	0.78 [0.67-0.92]**
**Educational attainment**				
No formal education	1,351 (38)	1,861 (36)	1.00	1.00
Up to primary	1,228 (27)	1,715 (28)	0.89 [0.76-1.04]	1.00 [0.84-1.19]
Up to secondary	1,405 (28)	2,084 (30)	0.91 [0.78-1.06]	1.07 [0.89-1.29]
Higher education	398 (7)	447 (6)	1.07 [0.83-1.38]	1.14 [0.84-1.55]
**Work status**				
Not working	3,152 (69)	4,161 (66)	1.00	1.00
Working	1,228 (31)	1,950 (34)	0.85 [0.75-0.97]*	0.94 [0.82-1.08]
**Asset quintile**				
Lowest	1,153 (34)	1,720 (36)	0.69 [0.56-0.86]***	0.72 [0.55-0.93]*
Lower	905 (23)	1,439 (25)	0.67 [0.53-0.84]***	0.73 [0.56-0.95]*
Middle	909 (19)	1,287 (19)	0.72 [0.57-0.91]**	0.76 [0.59-0.98]*
Higher	807 (14)	1,032 (13)	0.76 [0.60-0.98]*	0.82 [0.63-1.05]
Highest	621 (10)	640 (7)	1.00	1.00
**Place of residence**				
Urban	1,411 (22)	1,995 (24)	1.00	1.00
Rural	2,984 (78)	4,123 (76)	0.89 [0.76-1.01]	0.93 [0.81-1.06]
**HCP Advice**				
Not advised to quit tobacco by a HCP	3,807 (86)	4,533 (75)	1.00	1.00
Advised to quit tobacco by a HCP	588 (14)	1,585 (25)	0.48 [0.40-0.56]***	0.49 [0.42-0.59]***
**Cessation aid used**^**i**^				
Counseling/Cessation clinic	179 (3.66)	625 (8.87)	0.39 [0.30-0.50]***	0.43 [0.32-0.57]***
Nicotine replacement therapy	34 (0.44)	74 (0.94)	0.46 [0.24-0.87]*	0.70 [0.34-1.43]
Prescription medication	35 (0.61)	73 (0.80)	0.76 [0.40-1.43]	1.07 [0.47-2.39]
Ayurvedic, Homeopathic, Unani medicines^ii^	85 (1.97)	225 (2.92)	0.67 [0.43-1.02]*	0.93 [0.55-1.58]
Quitline	24 (0.59)	32 (0.54)	1.10 [0.48-2.51]	2.18 [0.74-6.44]

Legend:

Columns [2] and [3] show descriptive statistics of the individual sample from the Indian Global Adult Tobacco Survey (GATS) 2009–10 data, showing the sample (N) and survey-weighted frequency (%) of Attempting to Quit Tobacco among tobacco users who were successful and not successful in quitting, respectively.

Columns [4] and [5] show bivariate and fully adjusted odds ratios with 95% confidence intervals (ORs and 95% CI), respectively, from logistic regression analysis of successful compared to unsuccessful quitting amongst the 10,513 adults who attempted to quit tobacco from the Indian Global Adult Tobacco Survey (GATS) 2009–10 data.

^i^Each cessation aid has a reference category of non-use of that aid.

^ii^Ayurvedic, Homeopathic, Unani are traditional medicine practices widely used in India.

***p < 0.001, ** p < 0.01, * p < 0.05.

Being advised to quit tobacco by a HCP was associated with an adjusted odds of quitting 0.49 times (51%) lower than in those not advised by a HCP (95% CI 0.42-0.59, p <0.001). Use of cessation aids was also negatively associated with successful quit attempts. For example, counseling or counseling at the cessation clinic had an adjusted odds of quitting 0.43 times lower than using no cessation aids (95% CI 0.32-0.57, p <0.001). Use of prescription medication was associated with adjusted odds of quitting 1.07 times than not using any medication (95% CI 0.47-2.39, p = 0.87), however this was not statistically significant.

### Discussion

The study demonstrates strong associations between age, gender, educational attainment, and asset quintiles amongst adults who attempted to quit tobacco and were successful in quitting, which is also corroborated by other recent literature [22,23]. A study from Poland also found significant associations between older age, higher educational attainment, and being employed with quitting [22]. Male tobacco users had very high odds of quit attempts compared to females. This may be due to the fact that there were more male as compared to female tobacco users. We may also infer that although males made more quit attempts, they were less likely to be successful.

The effect of HCP advice was variable, with positive effects noted in persuading adults to attempt to quit but limited effects seen in assisting users in staying quit. The use of cessation aids, surprisingly, showed similar effects. The lack of benefit from HCP advice and use of cessation aids has been previously reported. In India, this may also be due to the lack of knowledge among HCPs about imparting tobacco cessation advice, and a study among doctors in the state of Kerala found lack of awareness and many missed opportunities in the patient-doctor interaction to impart tobacco cessation advice [24]. A systematic review of studies from developed countries was inconclusive regarding the benefit on quitting tobacco from being provided with intensive versus brief counseling [25]. Similarly, the benefits of long term follow-up and nicotine replacement therapy interventions produced marginal benefit with cessation outcomes, with no difference found between the two [25]. The lack of benefit seen in our study may be due to the fact that most tobacco users in India quit without the use of proven cessation modalities and this may be due to few existing cessation services and limited counseling facilities [15]. Our study was not designed to collect detailed information about the quality of cessation service, the duration of use and follow-up, all of which might help to explain why cessation services did not produce desired benefits. This type of information is also lacking in other countries [26], and these aspects of tobacco cessation deserve to be explored in subsequent research.

A further point is that tobacco use patterns in India cover a gamut of products such as cigarettes, bidis, hukkah, gutkha, paan with tobacco, khaini, and others [1]. The use of many of these products is socially and culturally acceptable, especially with respect to bidi smoking among men and chewing tobacco for women. Most of the evidence on quitting is from developed countries where tobacco use is predominantly in the form of cigarettes. Quitting each of the individual products used in India may require a different type of intervention. Further segregated analysis by type of tobacco product (smoked and smokeless) is required to explain these differences in quitting behavior.

This study has three important policy implications. First, the lower attempts to quit amongst the younger age groups suggest that if these users are not reached in time, India may be faced with an increased burden of tobacco-attributable diseases in the future. Poorer outcomes were also found in the less educated and lower asset quintile individuals, and thus these persons and the younger age groups will need specialized, targeted approaches to change their cessation behaviors and improve awareness levels. Second, with just over 10% of people attempting to quit using a cessation aid, there should be an increase in access to these services for at-risk populations, in order to assess their effectiveness. This requires that there be country-wide expansion and that services are more comprehensive with a larger number of cessation aids at each service site, including primary care centers, than is currently the case [26,27]. Third, the study confirms that almost 89% of tobacco users quit without the use of cessation therapies, and such quitting needs to be examined in detail vis-a-vis more resource intensive therapies such as NRT and behavioral interventions, which is consistent with other literature [28,29].

This study is the first of its kind in India that has analyzed nationally representative data on tobacco quit attempts. It is also the first national study that has examined correlates of quit attempts and successful quitting, especially by educational attainment and asset quintile as alternatives for wealth status or income. The strengths of the study are the national sample, the robust sampling methods used and the large number of persons interviewed [1]. However, the study is an analysis of secondary data, and as such it is bound by the limitations of those data. The study design also allowed the exploration of a limited number of variables. Variables such as duration and intensity of tobacco use and level of addiction were not available for all ever users. The study relied on self-reported, close-ended answers to questions on sensitive issues like tobacco use status, tobacco quit attempts, and utilization of cessation services, and there may have been some bias in responses as a result of social issues and perceived stigma. Furthermore, many of the responses were not validated by other more objective means, such as measurement of cotinine levels in those who said they had quit.

## Conclusions

This study provides the first national evidence on the relationships between quitting attempts and successful quitting with socio-demographic characteristics, health care provider advice and use of cessation services in India. The study highlights the need to provide cessation modalities amongst the younger, less educated, and poorer groups to prevent future tobacco-attributable diseases and widening health inequalities among these vulnerable groups. An increase in the coverage and access to cessation services is required, in order to comprehensively assess their effectiveness. Unassisted quitting without the use of cessation therapies seems to be quite successful in India at present, and such quitting also needs to be examined in more detail to understand the best cessation methods in light of constrained resources. This is particularly important to consider before prioritizing tobacco cessation services over the other approaches outlined in the National Tobacco Control Programme.

## Abbreviations

CI: Confidence interval; GATS: Global Adult Tobacco Survey; HCP: Health Care Provider; OR: Odds ratio.

## Competing interests

The authors’ declared that they have no competing interests.

## Authors’ contributions

SS, SM and ADH conceptualized and planned the study. SS led the data analysis, interpretation and writing of the manuscript. PL and MA contributed to the interpretation and writing of the manuscript. ADH and SM contributed to the interpretation and writing of the manuscript and provided the overall supervision. All authors read and approved the final manuscript.

## Pre-publication history

The pre-publication history for this paper can be accessed here:

http://www.biomedcentral.com/1471-2458/13/263/prepub
